# *Lactobacillus salivarius* reverse antibiotic-induced lung defense impairment in a ventilator model

**DOI:** 10.1186/s12967-018-1597-1

**Published:** 2018-08-13

**Authors:** Tzyy-Bin Tsay, Ming-Chieh Yang, Wan-Hsuan Chang, Pei-Hsuan Chen, Lee-Wei Chen

**Affiliations:** 1 Department of Surgery, Kaohsiung Armed Forces General Hospital Zuoying Branch, Kaohsiung, Taiwan; 2 Department of Surgery, Kaohsiung Veterans General Hospital, No. 386, Ta-chung 1st Road, Kaohsiung, 813 Taiwan; 3 Department of Biological Sciences, National Sun Yat-Sen University, No. 70, Lien-Hai Road, Kaohsiung, 804 Taiwan; 4 Institute of Emergency and Critical Care Medicine, National Yang-Ming University, No. 155, Sec. 2, Linong Street, Taipei, 112 Taiwan

**Keywords:** Mechanical ventilation, Reactive oxygen species, Alveolar macrophage, Peroxynitrite, Lung immunity

## Abstract

**Background:**

Widespread use of antibiotics in the intensive care unit is a potential cause of the emergence of hospital-acquired pneumonia. This study determined whether *Lactobacillus salivarius* feeding could reverse antibiotic-induced lung defense impairment in a ventilator model.

**Methods:**

C57BL/6 wild-type (WT) mice received mechanical ventilation for 3 h after intramuscular antibiotic treatment for 6 days. Treatment with dead *Lactobacillus salivarius* and fructo-oligosaccharides (FOS) feeding were used to stimulate antibacterial protein expression in the intestine. Reactive oxygen species (ROS) in the intestinal mucosa was detected using 2ʹ7ʹ-dichlorofluorescein diacetate. The peroxynitrite production of alveolar macrophages (AMs) was measured using dihydrorhodamine 123 oxidation assay. *N*-acetylcysteine (NAC), an ROS scavenger, was orally administered to mice receiving antibiotics with FOS feeding.

**Results:**

Antibiotic treatment decreased *Pseudomonas aeruginosa* (PA) phagocytic activity and activity of AMs and protein expression of regenerating islet-derived protein 3β (Reg3β) as well as Toll-like receptor 4 (TLR4) in the intestinal mucosa in the ventilator model. Antibiotic treatment also decreased ROS production in the intestinal mucosa, peroxynitrite production of AMs, and RELMβ expression as well as NF-κB DNA binding activity of the intestinal mucosa in WT mice but not in MyD88^−/−^ mice. Treatment with dead *L. salivarius* or FOS feeding increased ROS production, bacterial killing activity, and protein expression of Reg3β as well as TLR4 in the intestinal mucosa and reversed the inhibitory effects of antibiotics on PA phagocytic activity of AMs.

**Conclusion:**

Taken together with the finding that ablation of FOS-induced intestinal ROS using NAC decreased peroxynitrite production as well as PA phagocytic activity of AMs and protein expression of CRP-ductin, IL-17, Reg3β, and RELMβ in the intestinal mucosa, we conclude that commensal microflora plays a key role in stimulating lung immunity. Intestinal ROS plays a role as a predictive indicator and modulator of pulmonary defense mechanisms. Antibiotic treatment reduces lung defense against PA infection through the decrease in intestinal Reg3β and TLR4 expression. Treatment with dead *L. salivarius* or FOS feeding reverses the antibiotic-induced lung defense impairment through the intestinal ROS/MyD88 pathways.

## Background

Ventilator-associated pneumonia (VAP) remain to be a serious complication in critical patients receiving mechanical ventilation (MV) for > 48 h [1]. *Pseudomonas aeruginosa* (PA) is the most prevailing multidrug resistant (MDR) Gram-negative bacterium causing VAP [2]. It causes severe hospital-acquired infections, especially in an immunocompromized host, and is associated with a high mortality rate. Widespread use of antibiotics in the ICU is an important cause of the emergence of nosocomial infections caused by antibiotic-resistant Gram-negative bacteria [3, 4]. VAP is a common cause of death from nosocomial infection in ICU patients [5]. The prevalence of VAP ranges from 10 to 65%, with a high mortality rate, especially when it is induced by antibiotic-resistant bacteria [6, 7]. However, the effect and mechanism of antibiotic treatment on lung defense impairment remain undefined.

The emergence of antibiotic-resistant bacteria that are difficult or impossible to treat is becoming more and more common and causing a global health crisis. More than 2 million people are infected with antibiotic-resistant bacteria with 23,000 deaths in the United States every year [8]. Impairment of host defenses is a key element in the pathogenesis of antibiotic-resistant bacteria-induced infection. However, the mechanisms of antibiotic-mediated changes in host systemic defense in patients remain unclear.

The human GI tract is colonized by trillions of microorganisms that include hundreds of various species of viruses and bacteria [9, 10]. The normal microflora in the intestine act as a barrier against colonization and invasion of possibly pathogenic microorganisms. Recently, the spreading use of broad spectrum antibiotics such as ampicillin has led to elevated rates of colonization by potentially pathogenic members of *Enterobacteriaceae* such as *Klebsiella pneumoniae* [11]. Moreover, a retrospective cohort study of hospitalized patients showed that prior antibiotic treatment was closely associated with enhanced hospital mortality in patients with Gram-negative bacteremia complicated by severe sepsis. This may be owing to the enhanced antimicrobial resistance of the causative pathogens in patients receiving prior antibiotics [12]. Epithelial cells of the intestine express pattern recognition receptors (PRRs) that protect against microbial invasion while preserving epithelial barriers in the presence of commensal microflora [13]. The most recognized PRRs are the Toll-like receptors (TLRs). Paneth cells are critical contributors to restraint bacterial penetration through synthesis and release of a broad range of antimicrobial peptides and proteins such as enteric lysozyme, Reg3β, regenerating islet-derived protein 3γ (Reg3γ), RELMβ (resistin-like molecules β), α-defensins, cryptdin-related sequences (CRS) peptides, and CRP-ductin [14]. Reg3β is a bactericidal C-type lectin that is mainly produced in the gut that has antibacterial properties against Gram-negative bacteria [15]. Expression of Reg3γ is determined by TLR-MyD88-mediated signaling in the intestinal epithelial cells and is induced by commensal microbes [16]. However, the involvement of the gut–lung axis in VAP remains elusive. Most patients in the ICU are treated with antibiotics, which have pervasive and long duration effects on the intestinal microbiota [17]. We had earlier demonstrated that antibiotic treatment reduced the total number of bacteria in the terminal ileum, induced intestinal permeability, and increased the translocation of injected pathogenic *K. pneumonia* due to reduction in the mucosal bacterial killing activity and the expression of nondefensin proteins [18]. Recently, the intestinal microbiota has been recognized as a crucial player in the host defense system, supporting intestinal mucosal immunity and modulating systemic immunity [19–21]. The gut microbiota has been proven to enhance primary alveolar macrophage (AM) function and plays a protective role in the host defense against pneumococcal pneumonia [22]. We hypothesized that intestinal microbiota strengthens the host defense against PA-induced pneumonia in ventilator patients through reactive oxygen species (ROS) production and MyD88 signaling pathway in the intestine. In this study, antibiotic treatment with or without MV in mice was used as a model to study the effects and mechanisms of antibiotic treatment on lung defense mechanism. The primary objective of this study was to determine the effect of antibiotic treatment on lung defense in an MV model. Fructo-oligosacharides (FOS) are one of the most extensively studied prebiotics. *L. salivarius* is found in the mouth and the small intestine and is believed to play a role in enhancing the immune system [23]. The secondary objective was to determine whether feeding with dLac or fructooligosaccharides (FOS) could reverse the inhibitory effects of antibiotics on lung defense. The third objective was to examine the involvement of ROS as well as MyD88 signaling pathway in the intestinal mucosa in the inhibitory effect of antibiotic treatment on lung defense mechanism. In the future, using dLac or FOS to reverse antibiotic treatment-induced lung defense impairment in MV patients could be a useful therapeutic strategy.

## Methods

### Animals

Specific pathogen-free (SPF) C57BL/6J mice were obtained from the National Laboratory Breeding and Research Center (NLBRC, Taipei, Taiwan). Myd88^−/−^ (C57BL/6 background) mice were obtained from Oriental Bioservice, Inc (Kyoto, Japan). Experiments were in compliance with regulations approved by Kaohsiung Veterans General Hospital Animal Experiment Committee. All animal procedures were in compliance with the regulations on animals used for experimental and other scientific purposes approved by the Kaohsiung Veterans General Hospital Animal Experiments Committee.

## Experimental design

### Experiment 1

To simulate the use of combined antibiotic treatment in clinical patients with infections, we used a broad-spectrum antibiotic treatment regimen in which animals received a combined intramuscular injection of different antibiotics as follows: ampicillin (1000 mg/l; Bio Basic Inc.), vancomycin (500 mg/l; Hospira), and metronidazole (1000 mg/l; Sigma), 100 μl/day [18]. C57BL/6 mice were randomly divided into four groups as follows: Group I received intramuscular injection of normal saline for 6 days and sham operation; Group II received intramuscular injection of normal saline for 6 days and MV for 3 h; Group III received intramuscular injection of antibiotics for 6 days and sham operation; and Group IV received intramuscular injection of antibiotics for 6 days and MV for 3 h. Animals were sacrificed 3 h after MV or sham treatment. The lungs were harvested and assayed for neutrophil infiltration study. Phagocytic activity and TNF-α production of AMs were measured in another set of animals.

### Experiment 2

To examine the effect of prebiotic feeding on antibiotic treatment-induced lung defense impairment in the ventilator model, prebiotic FOS (Sigma) were given to mice (250 mg/day) to stimulate the growth of probiotic bacteria for 6 days. To further prove that the mechanisms for improvement and prevention by FOS supplementation are through increasing specific groups of intestinal commensal microbiota (*Lactobacillus* or *Bifidobacteria*), mice were fed with dead *Lactobacillus salivarius* CECT5713 [24] (2 × 10^8^ CFU/ml) for 6 days. C57BL/6 mice were randomly divided into five groups as follows: Group I received intramuscular injection of normal saline for 6 days and sham operation; Group II received intramuscular injection of normal saline for 6 days and MV for 3 h; Group III received intramuscular injection of antibiotics for 6 days and MV for 3 h; Group IV received antibiotics injection with FOS feeding for 6 days and MV for 3 h; and Group V received antibiotics injection with dead *Lactobacillus salivarius* CECT5713 [24] (2 × 10^8^ CFU/ml) feeding for 6 days and antibiotics injection for 6 days and MV for 3 h. The ileum was harvested for western blotting. Phagocytic activity and TNF-α production of AMs were measured in another set of animals.

### Experiment 3

To investigate the association between ROS production in the intestine and PA-induced neutrophil infiltration in the lung, FOS (250 mg/day) or dead *Lactobacillus salivarius* CECT5713 [24] (2 × 10^8^ CFU/ml) were given to mice for 6 days. C57BL/6 mice were randomly divided into four groups as follows: Group I received intramuscular injection of normal saline for 6 days; Group II received intramuscular injection of antibiotics for 6 days; Group III received intramuscular injection of antibiotics and FOS feeding for 6 days; and Group IV received intramuscular injection of antibiotics and dead *Lactobacillus salivarius* CECT5713 [24] (2 × 10^8^ CFU/ml) feeding for 6 days. ROS production of the intestine and peroxynitrite production of AMs were examined. Another set of animals received PA intratracheal instillation. Animals were sacrificed at 8 h after PA instillation and lungs were harvested for myeloperoxidase activity assay.

### Experiment 4

To examine the role of intestinal ROS production in antibiotic treatment-induced lung defense impairment; 0.1 mM *N*-acetylcysteine (NAC), an ROS scavenger, was orally administered to mice receiving antibiotics with FOS treatment for 3 days.

C57BL/6 mice were randomly divided into four groups as follows: Group I received intramuscular injection of normal saline for 6 days; Group II received intramuscular injection of antibiotics for 6 days; Group III received intramuscular injection of antibiotics and FOS feeding for 6 days; and Group IV received intramuscular injection of antibiotics with FOS feeding for 6 days and NAC feeding for 3 days. The production of 2′7′-dichlorofluorescein diacetate (DCFDA) and antibacterial protein expression in the ileum and phagocytic activity and peroxynitrite production of AMs were measured in all animals.

### FOS or dead *L. salivarius* feeding

To examine the effects of intestinal dysbiosis on lung defense mechanism, prebiotic (FOS, 250 mg/day; Sigma-Aldrich) or dead *L. salivarius* (CECT5713, 2 × 10^8^ CFU/ml) [24] was supplemented in drinking water to mice for 6 days (n = 50 in each group). Mice were given ad libitum access to water. The water with FOS or bacteria was changed every day. The dead bacteria were completely suspended in the water. There was no precipitation of bacteria in the bottle. The control group received drinking water without the supplementation of prebiotic or dead *L. salivarius.*

### Mechanical ventilation

Mice were anesthetized with Avertin (15 mg/kg, Sigma), and the neck was opened at 1 cm below the mouth. Muscles were divided and the trachea was cut and cannulated with 0.7 cm 21G flat syringe needle that connected to a mechanical ventilator (SAR-830/P, CWE, Pennsylvania, USA) for 3 h. During the duration of mechanical ventilation, the mice were administered Avertin (15 mg/kg) and sterile saline (10 μl/g) every hour. The ventilation protocol was high stretch (tidal volume = 30 ml/kg) and without end expiratory pressure (PEEP). The control mice breathed spontaneously during 3-h period.

### Neutrophil infiltration in the lungs

Lung myeloperoxidase (MPO) activity is a marker of lung neutrophil infiltration [25]. Lung tissues were harvested and homogenized in 50 mM potassium phosphate buffer (pH 6.0) with 0.5% hexadecyltrimethyl-ammonium bromide. Homogenates were centrifuged at 9500×*g*, 4 °C for 10 min. Supernatants (60 μl) was added to 939 μl of potassium phosphate buffer with 16.7 mg/ml of *O*-dianisidine and 0.5% hydrogen peroxide. The rate of change in absorbance at 460 nm was examined over 2 min. One unit of MPO activity is determined as the amount of enzyme that reduces 1 μmol of peroxide per min.

### Phagocytic activity of alveolar macrophages

Alveolar macrophages (AMs) represent the first line of lung defense against pathogens such as *P. aeruginosa* and epithelial cells promote the neutrophil sequestration into lungs which may cause lung injury as well as active the host immunity response [26]. The alveolar macrophages were collected and resuspended in HBSS as 10^6^ cells/ml. After 5 min of preincubation, the cell suspension was incubated with *P. aeruginosa* (10^8^/ml) at 37 °C for 1 h with shaking. The cells were removed as the pellet after centrifugation at 200×*g* for 10 min, and *P. aeruginosa* number in the supernatant was counted [27, 28].

### Ex vivo AMs stimulation

AMs were harvested from adult mice by bronchoalveolar lavage (BAL) with Tris-buffered saline containing 0.25 mM EDTA and EGTA. AMs were resuspended in RPMI 1640 in a final concentration of 1 × 10^5^ cells/ml. Cells were then cultured in 96-well microtiter plates for 2 h and washed with RPMI 1640 to remove non adherent cells [29]. Adherent monolayer cells were stimulated with 0.5 or 5 µg/ml of LPS (from *Escherichia coli* O26:B6 Sigma-Aldrich) or RPMI 1640 for 4 h. Supernatants were collected and stored at − 70 °C until assayed for TNF-α.

### Western immunoblots

The Reg3β, RELMβ, IL17, and CRP-ductin were identified by mouse monoclonal antibodies (*R&D Systems*); the TLR4 were identified by mouse monoclonal, rabbit polyclonal and goat polyclonal antibodies, respectively (Santa Cruz Biotechnology Inc.).

### Induction of *P. aeruginosa* pneumonia

Mice were anesthetized with xylazine (5 mg/kg intramuscularly, Bayer Inc., Mississauga, ON, Canada) and ketamine hydrochloride (100 mg/kg intramuscularly, Veterinary Laboratories, Wyeth-Ayerst Canada Inc., Mississauga, ON, Canada). The trachea was surgically opened and 50 µl (1.0 × 10^7^ CFU *P. aeruginosa*) were instilled via an angiocatheter through the trachea.

### ROS levels in the intestinal mucosa

The levels of ROS in the intestinal mucosa were analyzed by 10 mM DCFDA fluorescent dye (Sigma), which was added into the suspension of intestinal mucosa for cultivation. DCFDA is deacetylated by cellular esterases to a non-fluorescent compound, which is oxidized by ROS into 2′7′-dichlorofluorescein (DCF). DCF is detected by fluorescence spectroscopy with excitation and emission spectra of 495 and 529 nm, respectively [30].

### Peroxynitrite production of AMs (123-DHR oxidation assay of AMs)

Peroxynitrite is a potent macrophage-derived oxidizing cytotoxin that attacks invading pathogens. The collected AMs were adjusted to 3.0 × 10^6^ ml^−1^ in RPMI + 10% FBS, and 100 μl of HBSS without phenol red containing 25 μM of 1,2,3-dihydrorhodamine (Invitrogen, Eugene, OR), a peroxynitrite-detecting dye was added. The cells were stimulated with 1 μg/ml *Escherichia coli* LPS (Sigma). Peroxynitrite was determined using a fluorescence plate reader (Synergy HT Biotek, Winooski, VT) every 15 min for 75 min using excitation and emission wavelengths of 485 and 530 nm, respectively. Fluorescence due to auto-oxidation of 123-DHR was subtracted from the original data [31].

### Statistical analysis

All data were analyzed by one-way analysis of variance or T-test analysis of variance (ANOVA), followed by Turkey’s Multiple Comparison Test. All values in the figures and text were expressed as mean ± standard error of the mean. P values of less than 0.05 were considered to be statistically significant.

## Results

### MV treatment decreases PA phagocytic activity of AMs and antibiotic treatment further reduces it

**Fig. 1:**
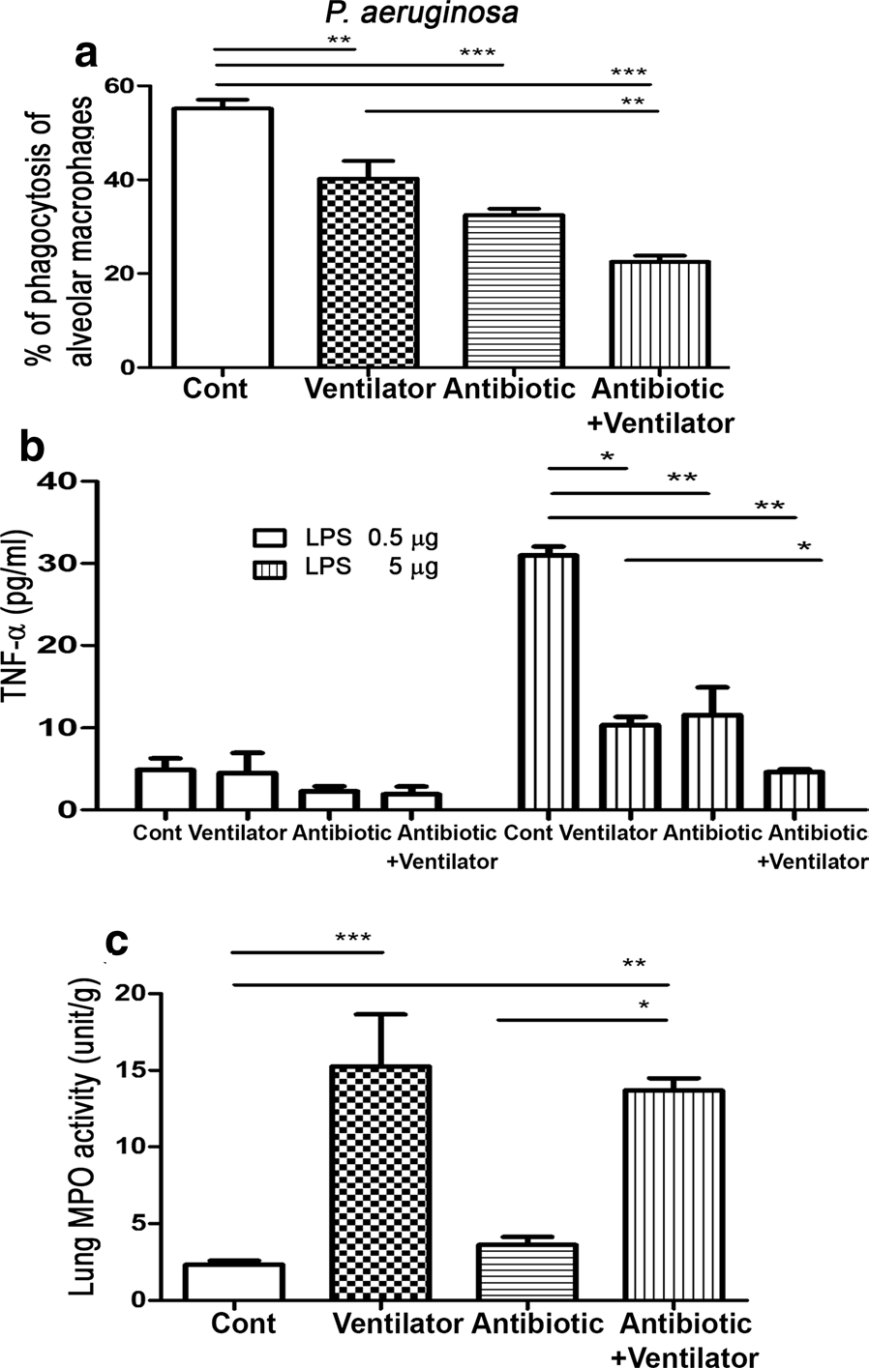
Effects of antibiotic treatment with or without MV on neutrophil infiltration in the lungs and PA phagocytic activity as well as activity of AMs. WT mice received intramuscular injection of combined antibiotic for 6 days and mechanical ventilation for 3 h and lung tissues were harvested and assayed. **a** MV decreased the PA phagocytic activity of AMs and antibiotic treatment for 6 days further decreased the PA phagocytic activity of AMs. AMs were collected and resuspended in HBSS as 10^6^ cells/ml. After 5 min of preincubation, the cell suspension was incubated with *P. aeruginosa* (10^8^/ml). The cells were removed and *P. aeruginosa* number in the supernatant was counted. Data are presented as mean ± SEM. **b** Antibiotic treatment for 6 days also significantly decreased activity of AMs. Antibiotic treatment before MV further decreased activity of AMs. AMs were harvested from adult mice by bronchoalveolar lavage (BAL). Cells were then cultured in 96-well microtiter plates to remove nonadherent cells. Adherent monolayer cells were stimulated with 0.5 or 5 µg/ml of LPS (from *Escherichia coli* O26:B6 Sigma-Aldrich). Supernatants were assayed for TNF-α. Data are presented as mean ± SEM. **c** Mechanical ventilation increased lung MPO activity. Antibiotic treatment did not change the MPO activity in sham or MV group. Data are presented as mean ± SEM; PA, *P. aeruginosa*; MPO: myeloperoxidase. *P < 0.05, **P < 0.01, ***P < 0.001

To study the effects of MV and antibiotics on lung defense mechanisms, AMs of WT mice were harvested for PA phagocytic activity assay after different treatments. MV significantly decreased PA phagocytic activity of AMs by 28% compared to that in the control group (40.27 ± 3.76 vs. 55.13 ± 1.96) (Fig. 1a). Antibiotic treatment for 6 days significantly decreased PA phagocytic activity of AMs by 42% compared to that in the control group (32.45 ± 1.4 vs. 55.13 ± 1.96). Moreover, antibiotic treatment with MV treatment further decreased the PA phagocytic activity of AMs by 44% compared to that in the MV group (22.5 ± 1.31 vs. 40.27 ± 3.76). These results indicate that MV treatment reduces PA phagocytic activity of AMs and antibiotic treatment further decreases it.

### MV treatment decreases activity of AMs and antibiotic treatment before MV further reduces it

To examine the mechanisms of antibiotic treatment-induced lung defense impairment, activity of AMs in WT mice was examined. MV treatment significantly decreased the activity of AMs by 66% compared to that in the control group (10.36 ± 0.97 vs. 30.99 ± 1.07 pg/ml) (Fig. 1b). Antibiotic treatment for 6 days significantly decreased the activity of AMs by 62% compared to that in the control group (11.58 ± 3.29 vs. 30.99 ± 1.07 pg/ml). Antibiotic treatment with MV treatment further decreased the activity of AMs by 55% compared to that in the MV group (4.62 ± 0.35 vs. 10.36 ± 0.97 pg/ml). These results indicate that MV treatment reduces the activity of AMs and antibiotic treatment before MV further decreases it.

### MV induces neutrophil infiltration in the lungs and antibiotics do not change it

To examine the effects of antibiotic or MV treatment on neutrophil infiltration in the lungs, pulmonary MPO activity was examined in the different groups. MV treatment significantly increased neutrophil infiltration in the lungs by sevenfold compared to that in the control group (15.26 ± 3.39 vs. 2.33 ± 0.25 unit/g) (Fig. 1c). Antibiotic treatment did not induce neutrophil infiltration in the lungs compared to that in the control group (3.63 ± 0.51 vs. 2.33 ± 0.25 unit/g). Antibiotic treatment before MV did not change lung MPO activity compared with that in the MV group. These results indicate that MV induces neutrophil infiltration in the lungs and antibiotic treatment does not change it.

### FOS or dead *L. salivarius* feeding reverses the inhibitory effect of antibiotics on PA phagocytic activity of AMs

**Fig. 2:**
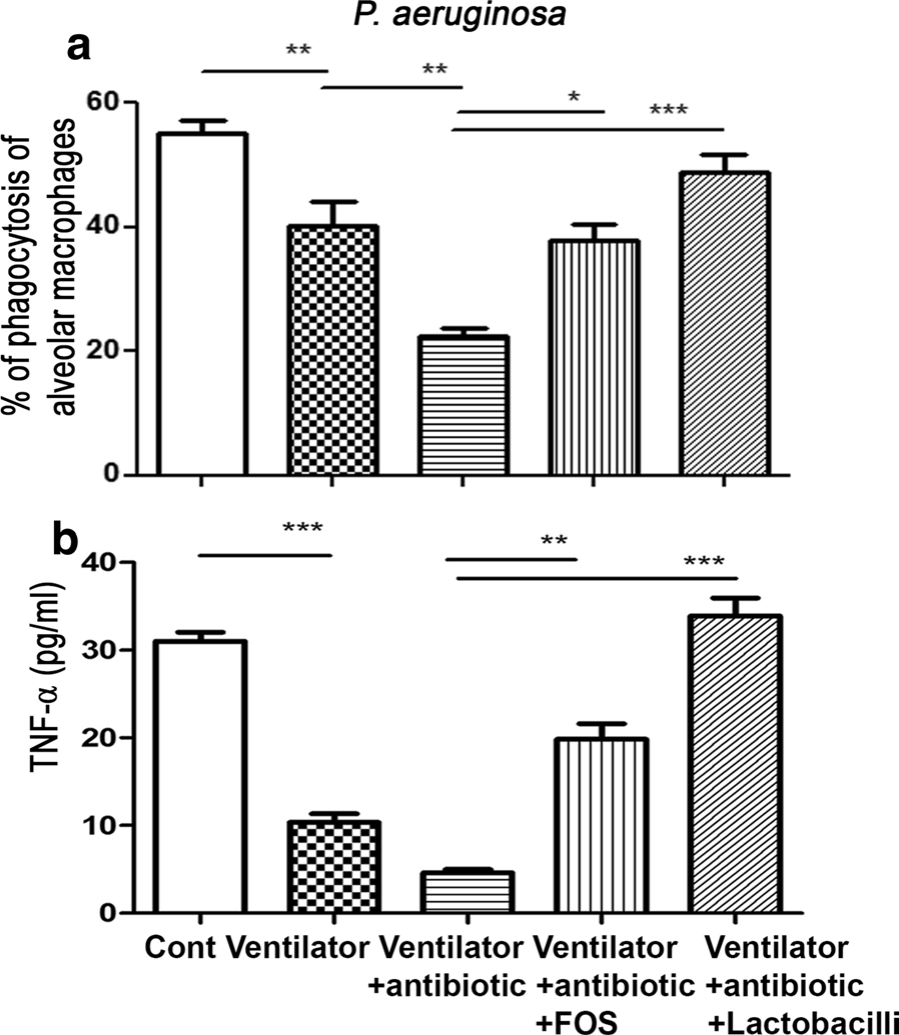
FOS or dead *L. salivarius* feeding reversed antibiotic-induced reduction of PA phagocytic activity and activity of AMs in the ventilator model. **a** Antibiotic treatment decreased the PA phagocytic activity of AMs in a ventilator model and FOS or dead *L. salivarius* feeding after antibiotic treatment reversed them. Data are presented as mean ± SEM. **b** Antibiotic treatment decreased activity of AMs in the ventilator model and FOS or dead *L. salivarius* feeding after antibiotic treatment reversed them. AMs were stimulated with LPS and supernatants were assayed for TNF-α. Data are presented as mean ± SEM.; PA, *P. aeruginosa*; FOS, fructo-oligosaccharides; dLac, dead *L. salivarius*. *P < 0.05, **P < 0.01

To assess whether probiotic or prebiotic treatment could reverse antibiotic treatment-induced PA phagocytic activity impairment of AMs, mice were fed with the prebiotic FOS or the probiotic dead *L. salivarius* before MV treatment. Oral feeding with FOS or dead *L. salivarius* significantly increased the PA phagocytic activity of AMs by 68% and 116%, respectively, compared to those in mice receiving MV after antibiotic treatment (37.8 ± 2.7 and 48.8 ± 2.8 vs. 22.5 ± 1.31) (Fig. 2a). These results suggest that FOS or dead *L. salivarius* feeding reverses the inhibitory effects of antibiotic treatment on PA phagocytic activity of AMs in the ventilator model.

### FOS or dead *L. salivarius* feeding restores the activity of AMs

To assess whether probiotic or prebiotic treatment could reverse antibiotic treatment-induced reduction of AM activity in the ventilator model, mice were fed with FOS or dead *L. salivarius*. Oral feeding of FOS or dead *L. salivarius* in mice receiving MV after antibiotic treatment significantly increased the activity of AMs to four- and sevenfold, respectively, compared to those in mice receiving antibiotics with MV treatment (19.87 ± 1.75 and 33.9 ± 2.06 vs. 4.62 ± 0.35) (Fig. 2b). These results suggest that FOS or dead *L. salivarius* feeding could reverse the inhibitory effects of antibiotic treatment on the activity of AMs.

### Antibiotics decrease Reg3β as well as TLR4 protein expression in the intestinal mucosa and FOS or dead *L. salivarius* feeding reverses them

**Fig. 3:**
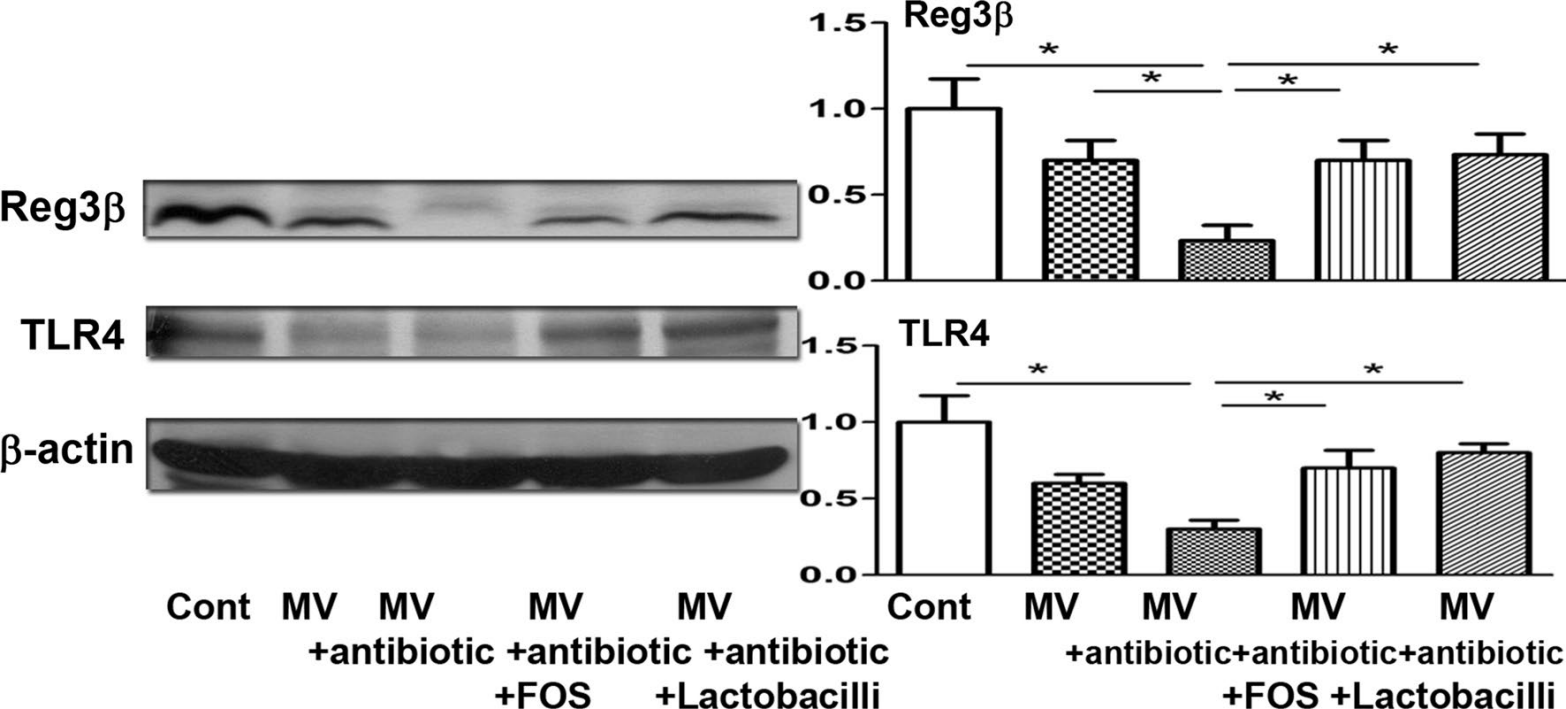
FOS or dead *L. salivarius* feeding reversed antibiotic-induced reduction of Reg3β and TLR4 expression in the intestinal mucosa in the ventilator model. Protein expression of Reg3β and TLR4 in the intestinal mucosa after different treatments was examined with Western blotting. Data are presented as mean ± SEM; PA, *P. aeruginosa*; FOS, fructo-oligosaccharides; dLac, dead *L. salivarius*. *P < 0.05

To examine the mechanisms of FOS or dead *L. salivarius* feeding on reversing antibiotic-induced lung defense impairment, the protein expression of Reg3β and TLR4 in the intestinal mucosa was examined. Antibiotic treatment before MV significantly decreased the protein expression of Reg3β and TLR4 in the intestinal mucosa compared to those in mice receiving MV (Fig. 3). FOS or dead *L. salivarius* feeding in mice receiving antibiotics with MV treatment significantly increased the protein expression of Reg3β and TLR4 in the intestinal mucosa compared to those in mice receiving antibiotics with MV treatment (Fig. 3). These results indicate that antibiotic treatment decreases the protein expression of Reg3β and TLR4 in the intestinal mucosa and feeding with FOS or dead *L. salivarius* restores them.

### Antibiotics decrease ROS levels in the intestinal mucosa and FOS or dead *L. salivarius* feeding restores them

**Fig. 4:**
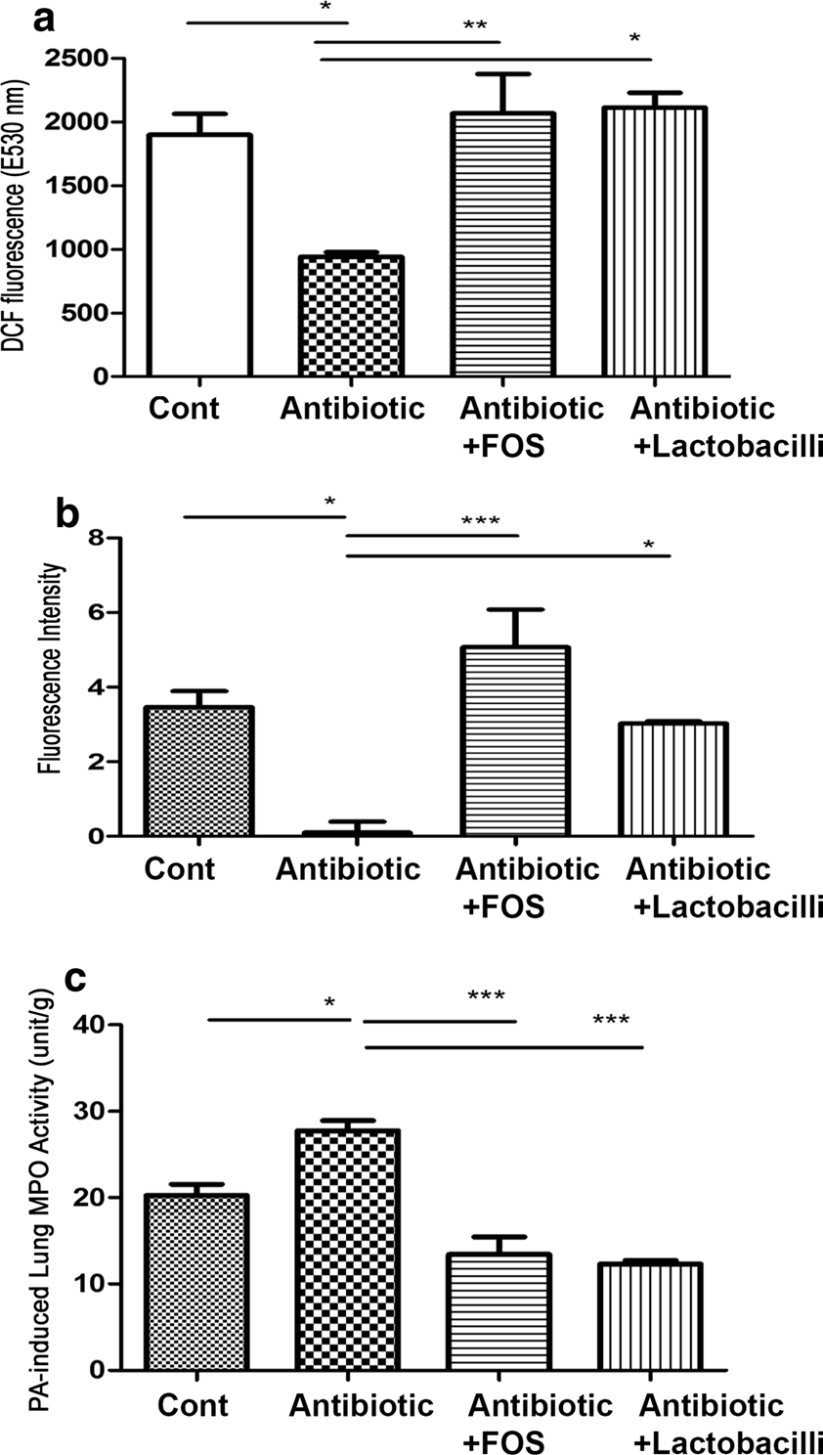
Antibiotic treatment decreased ROS production in the intestinal mucosa, peroxynitrite production of AMs and increased PA pneumonia-induced neutrophil infiltration in the lung. FOS or dead *L. salivarius* feeding reversed effects of antibiotic treatment. **a** Intramuscular combined antibiotic was given to mice for 6 days and ROS production in the intestinal mucosa was examined with the production of DCFDA. The levels of ROS in the intestinal mucosa were analyzed by DCFDA fluorescent dye, which was added into the suspension of intestinal mucosa for the cultivation. DCFDA is oxidized by ROS into 2ʹ7ʹ-dichlorofluorescein (DCF). DCF is detected by fluorescence spectroscopy with excitation and emission spectra of 495 and 529 nm, respectively. Data are presented as mean ± SEM. **b** Intramuscular combined antibiotic was given to mice for 6 days and AMs were purified from BALF for peroxynitrite production assay. The collected AMs were cultured with phenol red containing 25 μM of 1,2,3-dihydrorhodamine. The cells were stimulated with *E. coli* LPS. Peroxynitrite was measured every 15 min for 75 min using excitation and emission wavelengths of 485 and 530 nm, respectively. Data are presented as mean ± SEM. **c** Intramuscular combined antibiotic with or without FOS or dead *L. salivarius* feeding was given to mice for 6 days. The trachea of mice was surgically exposed and 50 µl (1.0 × 10^7^ CFU *P. aeruginosa*) were instilled via an angiocatheter through the trachea to induce *P. aeruginosa* pneumonia and neutrophil infiltration in the lungs was examined. Data are presented as mean ± SEM; PA, *P. aeruginosa*; DCF, 2ʹ7ʹ-dichlorofluorescein; FOS, fructo-oligosaccharides; MPO, myeloperoxidase; dLac, dead *L. salivarius*. *P < 0.05, **P < 0.01, ***P < 0.001

To examine the involvement of intestinal ROS in antibiotics-induced lung defense impairment, we examined the DCFDA levels in the intestinal mucosa. Antibiotic treatment significantly decreased the DCFDA levels in the intestinal mucosa by 52% compared to that in the control group (940.5 ± 34.61 vs. 1897 ± 163.8 E350 nm) (Fig. 4a). FOS or dead *L. salivarius* feeding in mice receiving antibiotic treatment significantly increased the DCFDA levels in the intestinal mucosa by twofold compared to those in the antibiotic treatment group (2065 ± 311.3 and 2111 ± 117.6 vs. 940.5 ± 34.61 E350 nm) (Fig. 4a). These results indicate that antibiotic treatment decreases ROS levels in the intestinal mucosa and FOS or dead *L. salivarius* feeding restores them.

### Antibiotics decrease peroxynitrite production of AMs and FOS or dead *L. salivarius* feeding restores them

Both superoxide and nitric oxide are essential components for the synthesis of microbicidal compounds in macrophages [31]. To further examine the mechanisms of antibiotic treatment-induced lung defense impairment, we harvested AMs from the lungs and examined the peroxynitrite production of AMs. Antibiotic treatment significantly decreased the peroxynitrite production of AMs by 97% compared to that in the control group (0.11 ± 0.02 vs. 3.46 ± 0.43) (Fig. 4b). Moreover, FOS or dead *L. salivarius* feeding in mice receiving antibiotic treatment significantly increased the peroxynitrite production of AMs compared to that in the antibiotic treatment group (5.07 ± 1.01 vs. 3.03 ± 0.06) (Fig. 4b). These results indicate that antibiotic treatment decreases peroxynitrite production of AMs and FOS or dead *L. salivarius* feeding restores it.

### Antibiotics enhance PA pneumonia-induced neutrophil infiltration in the lungs and FOS or dead *L. salivarius* feeding reverses it

Prior antibiotic treatment has been proven to increase patient mortality if Gram negative infection occurred [12]. To examine the effect of antibiotic treatment on PA pneumonia-induced neutrophil infiltration in the lungs, pulmonary MPO activity was assessed. Antibiotic treatment significantly increased 50% of PA pneumonia-induced MPO activity in the lungs compared to that in the PA injection group (27.76 ± 1.15 vs. 20.26 ± 1.3 unit/g) (Fig. 4c). Moreover, FOS or dead *L. salivarius* feeding reversed the stimulatory effect of antibiotic treatment on PA pneumonia-induced neutrophil infiltration in the lungs compared to that in the antibiotic treatment group (13.42 ± 2.04 and 12.31 ± 0.43 vs. 27.76 ± 1.15 unit/g) (Fig. 4c). These results indicate that antibiotic treatment enhances PA pneumonia-induced neutrophil infiltration in the lungs and FOS or dead *L. salivarius* feeding reverses it.

### NAC abolishes the stimulatory effect of FOS on antibacterial protein expression in the intestinal mucosa

**Fig. 5:**
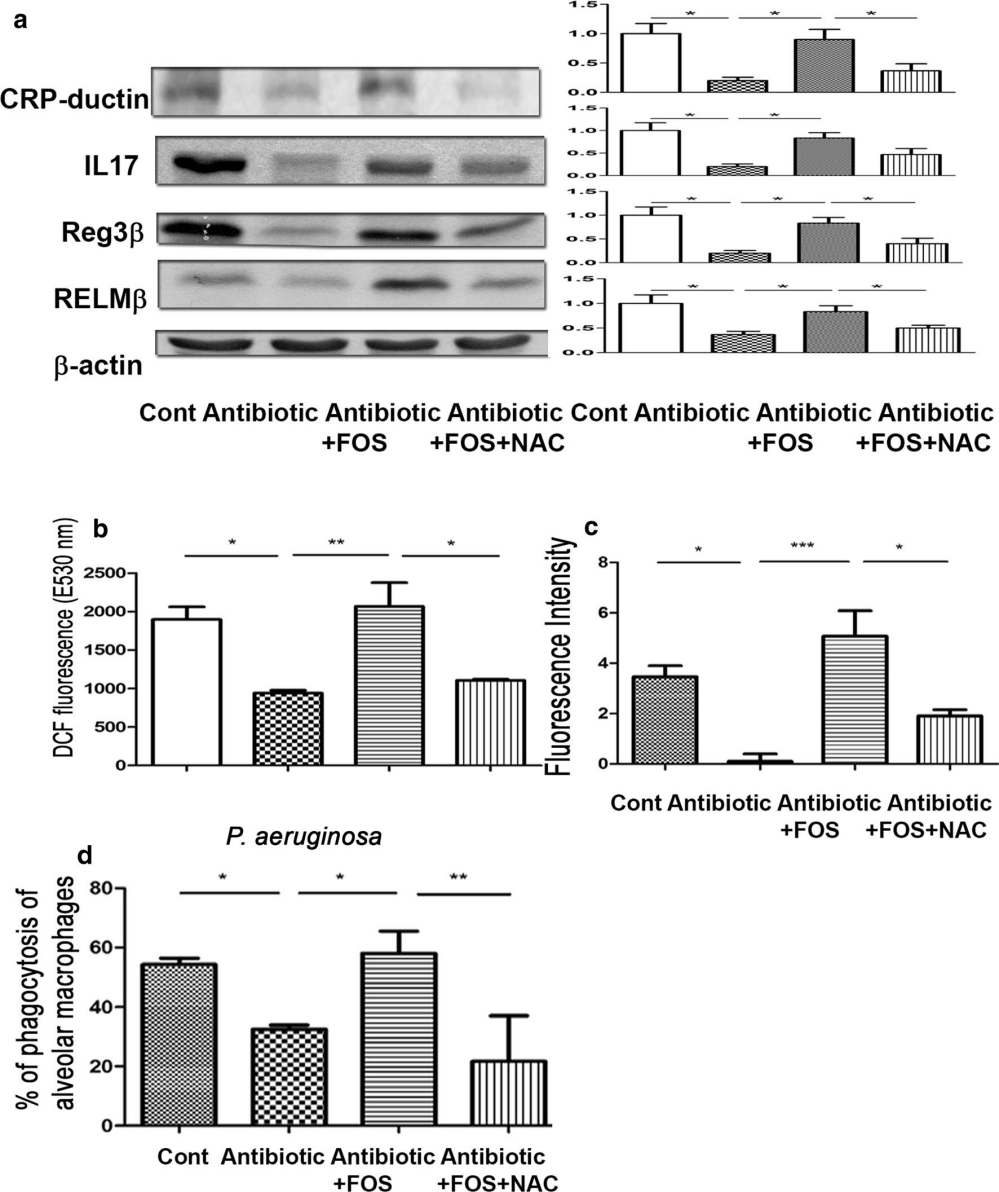
ROS scavenger, NAC, treatment abolishes the stimulatory effect of FOS feeding on antibacterial protein expression of the intestinal mucosa and reversed the stimulatory effect of FOS on DCFDA levels of the intestinal mucosa and peroxynitrite production as well as the PA phagocytic activity of AMs. **a** Antibiotic treatment before MV significantly decreased the protein expression of CRP-ductin, IL-17, Reg3β, and RELMβ of the intestinal mucosa as compared with the control group. FOS feeding in mice receiving antibiotic treatment significantly increased the protein expression of CRP-ductin, IL-17, Reg3β, and RELMβ of the intestinal mucosa as compared with the antibiotic group. NAC treatment reversed the stimulatory effect of FOS treatment on protein expression of CRP-ductin, IL-17, Reg3β, and RELMβ of the intestinal mucosa. Data are presented as mean ± SEM. **b** NAC, a ROS scavenger, was orally given to mice after antibiotics with FOS treatment to examine the involvement of intestinal ROS production in lung defense mechanisms. Mice received intramuscular injection of antibiotics with FOS feeding and NAC feeding for 3 days. ROS levels in the intestinal mucosa were analyzed by DCFDA fluorescent dye. DCF is detected by fluorescence spectroscopy with excitation and emission spectra of 495 nm and 529 nm, respectively. Data are presented as mean ± SEM. **c** NAC was given to mice after antibiotic with FOS treatment and AMs of those mice were harvested for peroxynitrite assay. Data are presented as mean ± SEM. **d** NAC was given to mice after antibiotic with FOS treatment and AMs of those mice were harvested for the PA phagocytic activity assay. Data are presented as mean ± SEM; PA, *P. aeruginosa*; DCF, 2ʹ7ʹ-dichlorofluorescein; FOS, fructo-oligosaccharides; NAC, *N*-acetylcysteine. *P < 0.05, **P < 0.01. FOS, fructo-oligosaccharides; NAC, *N*-acetylcysteine. *P < 0.05, **P < 0.01

To examine the involvement of intestinal ROS production in antibacterial protein expression in the intestine, the protein expression of CRP-ductin, IL-17, Reg3β, and RELMβ in the intestinal mucosa was examined. Antibiotic treatment significantly decreased the protein expression of CRP-ductin, IL-17, Reg3β, and RELMβ in the intestinal mucosa compared to that in the control group (Fig. 5a). FOS feeding in mice receiving antibiotic treatment significantly increased the protein expression of CRP-ductin, IL-17, Reg3β, and RELMβ in the intestinal mucosa compared to that in the antibiotic treatment group (Fig. 5a). Moreover, NAC treatment reversed the stimulatory effect of FOS treatment on the protein expression of CRP-ductin, IL-17, Reg3β, and RELMβ in the intestinal mucosa. These results indicate that FOS treatment reverses the inhibitory effect of antibiotic treatment on the protein expression of CRP-ductin, IL-17, Reg3β, and RELMβ in the intestinal mucosa. ROS production in the intestinal mucosa plays an important role in the stimulatory effect of FOS treatment on the antibacterial protein expression in the intestinal mucosa.

### NAC treatment decreases ROS levels in the intestinal mucosa

To examine the involvement of intestinal ROS production in antibiotic-induced lung defense impairment, DCFDA levels in the mucosa were examined in mice receiving antibiotics with FOS supplementation. NAC treatment in mice receiving antibiotics with FOS supplementation significantly decreased the DCFDA levels in the intestinal mucosa compared to those in mice receiving antibiotic with FOS treatment (1104.33 ± 78 vs. 2065.4 ± 311 E530 nm) (Fig. 5b). These results indicate that NAC treatment abolishes the stimulatory effects of FOS supplementation on ROS production in the intestinal mucosa.

### NAC treatment decreases peroxynitrite production of AMs

To further examine the involvement of intestinal ROS production in antibiotic-induced lung defense impairment, peroxynitrite production of AMs was examined. The stimulatory effect of FOS feeding on peroxynitrite production of AMs in mice receiving antibiotics was significantly decreased by NAC treatment compared to that in mice receiving antibiotics with FOS treatment (1.91 ± 0.2 vs. 5.07 ± 1.01) (Fig. 5c). These results indicate that oral feeding of ROS scavenger abolishes the stimulatory effect of FOS treatment on peroxynitrite production of AMs and ROS production in the intestine plays a crucial role in lung defense mechanisms.

### NAC abolishes the stimulatory effect of FOS on PA phagocytic activity of AMs

To further examine the mechanism of intestinal ROS production in antibiotic-induced lung defense impairment, phagocytic activity of AMs was examined. The stimulatory effect of FOS feeding after antibiotic treatment on PA phagocytic activity of AMs was abolished by NAC treatment compared to that in mice receiving antibiotics with FOS treatment (21.65 ± 15.35 vs. 58.03 ± 7.47) (Fig. 5d). These results indicate that oral feeding with intestinal ROS scavenger abolishes the stimulatory effect of FOS treatment on PA phagocytic activity of AMs and intestinal ROS plays an important role in the phagocytic activity of AMs.

### Antibiotic treatment does not change ROS levels in the intestine of MyD88^−/−^ mice

**Fig. 6:**
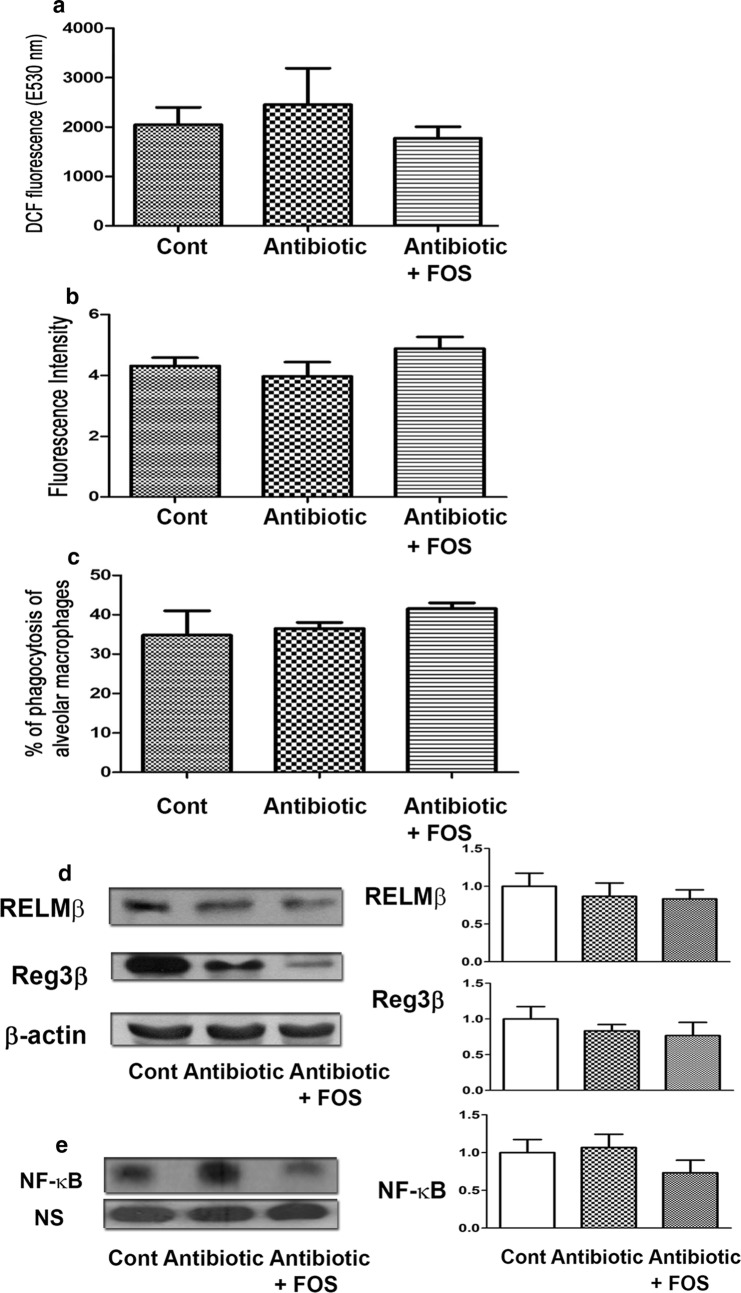
Antibiotic treatment does not change antibacterial protein expression as well as ROS production of intestine and peroxynitrite production as well as phagocytic activity of AMs in MyD88^−**/**−^ mice. **a** Antibiotic treatment decrease DCFDA levels of the intestinal mucosa in WT mice but not in MyD88^−/−^ mice. FOS feeding after antibiotic treatment did not change DCFDA levels of the intestinal mucosa in MyD88^−**/**−^ mice as compared with the antibiotic group. The levels of reactive oxygen species in the intestinal mucosa were analyzed by DCFDA fluorescent dye. DCF is detected by fluorescence spectroscopy with excitation and emission spectra of 495 and 529 nm, respectively. Data are presented as mean ± SEM. **b** Antibiotic treatment did not change the peroxynitrite production of AMs in MyD88^−**/**−^ mice as compared with the control group. FOS treatment after antibiotic did not change the peroxynitrite production of AMs in MyD88^−**/**−^ mice as compared with the antibiotic group. Data are presented as mean ± SEM. **c** Antibiotic treatment did not change the PA phagocytic activity of AMs in MyD88^−**/**−^ mice as compared with the control group. FOS treatment after antibiotic did not change PA phagocytic activity of AMs in MyD88^−**/**−^ mice as compared with the antibiotic group. Data are presented as mean ± SEM. **d** Antibiotic treatment did not change RELMβ and Reg3β of the intestinal mucosa in MyD88^−/−^ mice. FOS treatment after antibiotic did not change RELMβ and Reg3β in MyD88^−**/**−^ mice. Data are presented as mean ± SEM. **e** Antibiotic treatment did not change NF-κB DNA binding activity of the intestinal mucosa in MyD88^−**/**−^ mice as compared with the control group. FOS treatment after antibiotic did not change NF-κB DNA binding activity in MyD88^−**/**−^ mice as compared with the antibiotic group. Data are presented as mean ± SEM; DCF, 2ʹ7ʹ-dichlorofluorescein; FOS, fructo-oligosaccharides

To examine the role of MyD88 in antibiotic-induced reduction of intestinal ROS production, DCFDA levels in the intestinal mucosa of MyD88^−**/**−^ mice were examined. Antibiotic treatment decrease the DCFDA levels in the intestinal mucosa in WT mice but not in MyD88^−**/**−^ mice. Moreover, FOS feeding after antibiotic treatment did not change DCFDA levels in the intestinal mucosa in MyD88^−**/**−^ mice compared to those in the antibiotic treatment group (Fig. 6a). These results indicate that MyD88 plays a key role in the inhibitory effects of antibiotic treatment on intestinal ROS production.

### Antibiotic treatment does not change peroxynitrite production of AMs in MyD88^−/−^ mice

To examine the involvement of MyD88 in the inhibitory effect of antibiotic treatment on the lung defense mechanisms, peroxynitrite production of AMs in MyD88^−**/**−^ mice was examined. Antibiotic treatment did not change the peroxynitrite production of AMs in MyD88^−**/**−^ mice compared to that in the control group. Furthermore, FOS treatment after antibiotic treatment did not change the peroxynitrite production of AMs in MyD88^−**/**−^ mice compared to that in the antibiotic treatment group (Fig. 6b). These results indicate that MyD88 plays an important role in the inhibitory effect of antibiotic treatment on peroxynitrite production of AMs.

### Antibiotic treatment does not change PA phagocytic activity of AMs in MyD88^−/−^ mice

To examine the role of MyD88 in the inhibitory effect of antibiotic treatment on the lung defense mechanisms, PA phagocytic activity of AMs in MyD88^−**/**−^ mice was examined. Antibiotic treatment did not change PA phagocytic activity of AMs in MyD88^−**/**−^ mice compared to that in the control group. Furthermore, FOS treatment after antibiotic treatment did not change PA phagocytic activity of AMs in MyD88^−**/**−^ mice compared to that in the antibiotic treatment group (Fig. 6c). These results indicate that MyD88 plays an important role in the effects of antibiotic treatment with or without FOS treatment on PA phagocytic activity of AMs.

### Antibiotic treatment does not change the protein expression of Reg3β and RELMβ in the intestinal mucosa in MyD88^−/−^ mice

To examine the role of MyD88 in the inhibitory effect of antibiotic treatment on antibacterial protein expression in the intestinal mucosa, the protein expression of Reg3β and RELMβ in the intestinal mucosa was examined. Antibiotic treatment did not change the expression of Reg3β and RELMβ in the intestinal mucosa in MyD88^−**/**−^ mice compared to that in the control group. Furthermore, FOS treatment after antibiotic treatment did not change the expression of Reg3β and RELMβ in MyD88^−**/**−^ mice compared to that in the antibiotic treatment group (Fig. 6d). These results indicate that MyD88 plays an important role in the inhibitory effects of antibiotic treatment on the protein expression of Reg3β and RELMβ in the intestinal mucosa.

### Antibiotic treatment does not change NF-κB DNA binding activity of the intestinal mucosa in MyD88^−/−^ mice

Antibiotic treatment did not change NF-κB DNA binding activity of the intestinal mucosa in MyD88^−/−^ mice compared to that in the control group. Furthermore, FOS treatment after antibiotic treatment did not change NF-κB DNA binding activity in MyD88^−**/**−^ mice compared to that in the antibiotic treatment group (Fig. 6e). These results indicate that MyD88 plays an important role in the inhibitory effects of antibiotics on NF-κB DNA binding activity of the intestinal mucosa.

## Discussion

VAP is the main cause of nosocomial infection in the ICU and a risk factor for increased mortality. MV has been known to decrease lung defense mechanisms through the increase of ROS production of AMs [32]. MV also induces the lung inflammatory injury through the NLRP3 inflammasome of AMs [33]. However, the effect of antibiotic treatment on the lung defense mechanism in a ventilator model has not been well characterized. We used an antibiotic treatment in mice to study the effect and mechanism of antibiotic treatment on lung defense mechanism. First, MV decreased the activity and PA phagocytic activity of AMs and increased the neutrophil infiltration in the lungs. Antibiotic treatment for 6 days decreased the PA phagocytic activity of AMs. Antibiotic treatment before MV further decreased the PA phagocytic activity compared to that in the MV group. These results indicate that MV treatment decreases the lung defense mechanism and antibiotic treatment before MV further decreases it. Furthermore, MV treatment significantly decreased the activity of AMs. Antibiotic treatment before MV further decreased the activity of AMs. To examine the mechanism of antibiotic treatment in the lung defense mechanism, we examined the MPO activity in the lung. Ventilator alone significantly increased the MPO activity in the lung. Antibiotic treatment did not increase the MPO activity in the lung. Antibiotic treatment before MV did not further increase the MPO activity in the lungs compared to that in the MV group. These results indicate that antibiotic treatment induces lung defense impairment through the reduction of bacteria phagocytic activity of AMs but not the neutrophil infiltration in the lung. MV and antibiotic treatment induce AM phagocytic activity impairment through different mechanisms. Our results highlight the possibility that broad-spectrum antibiotics may diminish innate immune defense against PA infection in the lungs.

Daily administration of living *L. salivarius* to healthy adults has been proved to be safe and improve gut microbiota and different parameters related to immune response [24]. To decrease the confounding factors of antibiotic treatment on living *L. salivarius*, we fed antibiotic treatment animals with dead *L. salivarius* instead of living *L. salivarius* to enhance the immune system of intestinal tract. Previously, living *L. salivarius* have been proved to provide anti-inflammatory effect on TNBS-induced colonic damage in a rat model but not the dead strain [34]. Our data demonstrated that oral feeding with dead *L. salivarius* restored the protein expression of Reg3β and TLR4 in the intestinal mucosa and reversed the antibiotic-induced reduction of PA phagocytic activity of AMs. Therefore, we speculate that living *L. salivarius* will provide better enhancing effects on host defense mechanisms in the antibiotic treatment before MV model than the dead strain.

Intestinal microbes not only contribute to the local defense against infections, but also modulate systemic immune response especially the lung [34]. Recent breakthroughs in the understandings of the involvement of the gut microbiome in both health and disease have critical implications for respiratory and critical care medicine [22]. An earlier finding suggested that *Lactobacillus rhamnosus* administration is safe in critically ill patients and efficacious for the prevention of VAP [34]. To assess whether probiotic or prebiotic treatment could reverse antibiotic treatment-induced reduction of PA phagocytic activity of AMs in a ventilator model, mice were fed with the prebiotic FOS or the probiotic dead *L. salivarius*. We clearly demonstrated that the gut microbiota plays a protective role in PA-induced VAP as evidenced by the decrease in the phagocytic activity and activity of AMs in mice treated with antibiotics. Oral feeding with FOS or dead *L. salivarius* in mice receiving antibiotic treatment before MV significantly increased the PA phagocytic activity of AMs. These results indicate that FOS or dead *L. salivarius* feeding reverses the inhibitory effects of antibiotic treatment on lung defense mechanism in the mice ventilator model. To examine the involvement of intestinal TLRs as well as antibacterial protein expression in the stimulatory effects of FOS or dead *L. salivarius* feeding on antibiotic-induced lung defense impairment, the protein expression of Reg3β and TLR4 in the intestinal mucosa was examined. Antibiotic treatment significantly decreased the protein expression of Reg3β and TLR4 in the intestinal mucosa compared to that in the MV group. FOS or dead *L. salivarius* feeding in mice receiving MV after antibiotic treatment significantly increased the protein expression of Reg3β and TLR4 in the intestinal mucosa compared to that in the antibiotic treatment before MV group. Together, our data suggest that antibiotic treatment decreases the protein expression of Reg3β and TLR4 in the intestinal mucosa in the mice ventilator model. Oral feeding with FOS or dead *L. salivarius* restores the protein expression of Reg3β and TLR4 in the intestinal mucosa and reverses the antibiotic-induced reduction of PA phagocytic activity of AMs. Our studies provide a basis for the development of a novel therapeutic strategy for VAP.

ROS levels in the gut have been suggested to be closely related to the intestinal barrier function [34]. Out data demonstrated that antibiotic treatment significantly decreased the DCFDA levels in the intestinal mucosa compared to those in the control group. FOS or dead *L. salivarius* feeding in mice receiving antibiotic treatment significantly increased the DCFDA levels in the intestinal mucosa. These results indicate that antibiotic treatment decreases intestinal ROS levels and FOS or dead *L. salivarius* feeding restores them. To examine the mechanisms of antibiotic treatment in the lung defense, we further examined the peroxynitrite production of AMs. MV has been previously shown to decrease lung defense mechanisms through the increase of ROS production of AMs [32]. Our data demonstrated that antibiotic treatment significantly decreased the peroxynitrite production of AMs compared to that in the control group. Moreover, oral feeding with FOS or dead *L. salivarius* in mice receiving antibiotic treatment significantly increased the peroxynitrite production of AMs compared to that in mice treated with antibiotics. These results indicate that antibiotic treatment decreases lung defense of AMs and FOS or dead *L. salivarius* feeding restores it. Furthermore, our data demonstrated that antibiotic treatment before PA intratracheal injection significantly increased PA pneumonia-induced neutrophil infiltration and FOS or dead *L. salivarius* feeding reversed the stimulatory effect of antibiotic treatment on PA pneumonia-induced neutrophil infiltration in the lungs. To further examine the involvement of intestinal ROS production in lung defense mechanisms, NAC, a ROS scavenger, was orally given to mice after FOS treatment. The stimulatory effect of FOS feeding on ROS production in the intestinal mucosa was abolished by NAC treatment compared to that in the antibiotic + FOS treatment group. The stimulatory effect of FOS feeding after antibiotic treatment on peroxynitrite production and the PA phagocytic activity of AMs were also abolished by NAC treatment. In summary, antibiotic treatment induces lung defense mechanism impairment through the decrease of ROS production of the gut mucosa, the peroxynitrite production of AMs, and the subsequent phagocytic activity of AMs. These results indicate that oral feeding with the intestinal ROS scavenger inhibits the stimulatory effect of FOS feeding on the lung defense mechanism by decreasing the intestinal ROS production and peroxynitrite production of AMs. Our data identify a gut–lung axis in VAP and establish a mechanism for pulmonary immunomodulation by the intestinal mucosa.

We further examine the role of intestinal antibacterial protein in the stimulatory effect of FOS treatment on lung defense mechanism. Antibiotic treatment significantly decreased the protein expression of CRP-ductin, IL-17, Reg3β, and RELMβ in the intestinal mucosa. FOS or dead *L. salivarius* feeding in mice receiving MV after antibiotic treatment significantly increased the protein expression of Reg3β and TLR4 in the intestinal mucosa compared to that in the antibiotic treatment with MV group. NAC treatment after FOS feeding significantly decreases FOS-induced CRP-ductin, IL-17, Reg3β, and RELMβ expression in the mucosa. NAC treatment could abolish the stimulatory effect of ROS on intestinal antibacterial protein expression and peroxynitrite production of AMs. These results suggest that ROS production in the intestinal mucosa plays an important role in the stimulatory effect of FOS feeding on the antibacterial protein expression in the intestinal mucosa.

Commensal microflora has been known to maintain systemic defense mechanisms. An earlier study showed that MyD88 is an important signaling pathway between bacteria and host defense mechanism [34]. Therefore, we examined the involvement of MyD88 in antibiotic treatment with or without FOS supplementation in the lung defense mechanism. Antibiotic treatment did not decrease the ROS production in the intestinal mucosa, the peroxynitrite production of AMs, and the PA phagocytic activity of AMs in MyD88^−**/**−^ mice. Moreover, antibiotic treatment did not decrease Reg3β and RELMβ protein expression in the intestinal mucosa in MyD88^−**/**−^ mice. Furthermore, FOS treatment after antibiotic treatment did not increase DCFDA levels and intestinal Reg3β as well as RELMβ protein expression, peroxynitrite production of AMs, and PA phagocytic activity of AMs in MyD88^−**/**−^ mice. Altogether, these results indicate that antibiotic treatment decreases DCFDA levels in the intestinal mucosa, peroxynitrite production of AMs, and PA phagocytic activity of AMs through the MyD88 signaling pathway.

## Conclusions

Antibiotic treatment decreases the protein expression of TLR4, CRP-ductin, IL-17, Reg3β, and RELMβ and ROS production in the intestinal mucosa. The decrease in ROS production in the intestinal mucosa induces the decrease in peroxynitrite production as well as phagocytic activity of AMs. FOS or dLac feeding increase ROS production and stimulates the protein expression of TLR4, CRP-ductin, IL-17, Reg3β, and RELMβ in the intestinal mucosa and peroxynitrite production as well as phagocytic activity of AMs. FOS treatment reverses the antibiotic-induced lung defense mechanism through MyD88 signaling. Commensal microflora plays an important role in stimulating lung defense through the induction of intestinal ROS production. These observations imply that oral feeding with a TLR4 stimulator may be useful to increase the lung defense mechanism in patients undergoing MV with antibiotic treatment.

## References

[1] Valencia M, Torres A (2009). Ventilator-associated pneumonia. Curr Opin Crit Care.

[2] El Solh AA, Alhajhusain A (2009). Update on the treatment of *Pseudomonas aeruginosa* pneumonia. J Antimicrob Chemother.

[3] Fagon JY, Chastre J, Domart Y, Trouillet JL, Pierre J, Darne C, Gibert C (1989). Nosocomial pneumonia in patients receiving continuous mechanical ventilation. Prospective analysis of 52 episodes with use of a protected specimen brush and quantitative culture techniques. Am Rev Respir Dis.

[4] Kollef MH, Vlasnik J, Sharpless L, Pasque C, Murphy D, Fraser V (1997). Scheduled change of antibiotic classes: a strategy to decrease the incidence of ventilator-associated pneumonia. Am J Respir Crit Care Med.

[5] Vincent JL, Bihari DJ, Suter PM, Bruining HA, White J, Nicolas-Chanoin MH, Wolff M, Spencer RC, Hemmer M (1995). The prevalence of nosocomial infection in intensive care units in Europe. Results of the European Prevalence of Infection in Intensive Care (EPIC) Study. EPIC International Advisory Committee. JAMA.

[6] Rello J, Torres A, Ricart M, Valles J, Gonzalez J, Artigas A, Rodriguez-Roisin R (1994). Ventilator-associated pneumonia by *Staphylococcus aureus*. Comparison of methicillin-resistant and methicillin-sensitive episodes. Am J Respir Crit Care Med.

[7] Meyer KS, Urban C, Eagan JA, Berger BJ, Rahal JJ (1993). Nosocomial outbreak of Klebsiella infection resistant to late-generation cephalosporins. Ann Intern Med.

[8] Hampton T (2013). Report reveals scope of US antibiotic resistance threat. JAMA.

[9] Antonopoulos DA, Huse SM, Morrison HG, Schmidt TM, Sogin ML, Young VB (2009). Reproducible community dynamics of the gastrointestinal microbiota following antibiotic perturbation. Infect Immun.

[10] Ley RE, Lozupone CA, Hamady M, Knight R, Gordon JI (2008). Worlds within worlds: evolution of the vertebrate gut microbiota. Nat Rev Microbiol.

[11] Parm U, Metsvaht T, Sepp E, Ilmoja ML, Pisarev H, Pauskar M, Lutsar I (2010). Impact of empiric antibiotic regimen on bowel colonization in neonates with suspected early onset sepsis. Eur J Clin Microbiol Infect Dis.

[12] Johnson MT, Reichley R, Hoppe-Bauer J, Dunne WM, Micek S, Kollef M (2011). Impact of previous antibiotic therapy on outcome of Gram-negative severe sepsis. Crit Care Med.

[13] Dupaul-Chicoine J, Yeretssian G, Doiron K, Bergstrom KS, McIntire CR, LeBlanc PM, Meunier C, Turbide C, Gros P, Beauchemin N (2010). Control of intestinal homeostasis, colitis, and colitis-associated colorectal cancer by the inflammatory caspases. Immunity.

[14] Meyer-Hoffert U, Hornef MW, Henriques-Normark B, Axelsson LG, Midtvedt T, Putsep K, Andersson M (2008). Secreted enteric antimicrobial activity localises to the mucus surface layer. Gut.

[15] Miki T, Holst O, Hardt WD (2012). The bactericidal activity of the C-type lectin RegIIIbeta against Gram-negative bacteria involves binding to lipid A. J Biol Chem.

[16] Brandl K, Plitas G, Mihu CN, Ubeda C, Jia T, Fleisher M, Schnabl B, DeMatteo RP, Pamer EG (2008). Vancomycin-resistant enterococci exploit antibiotic-induced innate immune deficits. Nature.

[17] Schuijt TJ, van der Poll T, de Vos WM, Wiersinga WJ (2013). The intestinal microbiota and host immune interactions in the critically ill. Trends Microbiol.

[18] Wu YY, Hsu CM, Chen PH, Fung CP, Chen LW (2014). Toll-like receptor stimulation induces nondefensin protein expression and reverses antibiotic-induced gut defense impairment. Infect Immun.

[19] Trompette A, Gollwitzer ES, Yadava K, Sichelstiel AK, Sprenger N, Ngom-Bru C, Blanchard C, Junt T, Nicod LP, Harris NL, Marsland BJ (2014). Gut microbiota metabolism of dietary fiber influences allergic airway disease and hematopoiesis. Nat Med.

[20] Clemente JC, Ursell LK, Parfrey LW, Knight R (2012). The impact of the gut microbiota on human health: an integrative view. Cell.

[21] Caballero S, Pamer EG (2015). Microbiota-mediated inflammation and antimicrobial defense in the intestine. Annu Rev Immunol.

[22] Schuijt TJ, Lankelma JM, Scicluna BP, de Melo SF, Roelofs JJ, de Boer JD, Hoogendijk AJ, de Beer R, de Vos A, Belzer C (2016). The gut microbiota plays a protective role in the host defence against pneumococcal pneumonia. Gut.

[23] O’Hara AM, O’Regan P, Fanning A, O’Mahony C, Macsharry J, Lyons A, Bienenstock J, O’Mahony L, Shanahan F (2006). Functional modulation of human intestinal epithelial cell responses by *Bifidobacterium infantis* and *Lactobacillus salivarius*. Immunology.

[24] Sierra S, Lara-Villoslada F, Sempere L, Olivares M, Boza J, Xaus J (2010). Intestinal and immunological effects of daily oral administration of *Lactobacillus salivarius* CECT5713 to healthy adults. Anaerobe.

[25] Liu YY, Liao SK, Huang CC, Tsai YH, Quinn DA, Li LF (2009). Role for nuclear factor-kappaB in augmented lung injury because of interaction between hyperoxia and high stretch ventilation. Transl Res.

[26] Sibille Y, Reynolds HY (1990). Macrophages and polymorphonuclear neutrophils in lung defense and injury. Am Rev Respir Dis.

[27] Sayek I (1997). Animal models for intra-abdominal infection. Hepatogastroenterology.

[28] van Westerloo DJ, Weijer S, Bruno MJ, de Vos AF, Van’t Veer C, van der Poll T (2005). Toll-like receptor 4 deficiency and acute pancreatitis act similarly in reducing host defense during murine *Escherichia coli* peritonitis. Crit Care Med.

[29] Renckens R, van Westerloo DJ, Roelofs JJ, Pater JM, Schultz MJ, Florquin S, van der Poll T (2008). Acute phase response impairs host defense against *Pseudomonas aeruginosa* pneumonia in mice. Crit Care Med.

[30] Esparza A, Gerdtzen ZP, Olivera-Nappa A, Salgado JC, Nunez MT (2015). Iron-induced reactive oxygen species mediate transporter DMT1 endocytosis and iron uptake in intestinal epithelial cells. Am J Physiol Cell Physiol.

[31] Allen RG, Lafuse WP, Galley JD, Ali MM, Ahmer BM, Bailey MT (2012). The intestinal microbiota are necessary for stressor-induced enhancement of splenic macrophage microbicidal activity. Brain Behav Immun.

[32] Kallet RH, Matthay MA (2013). Hyperoxic acute lung injury. Respir Care.

[33] Wu J, Yan Z, Schwartz DE, Yu J, Malik AB, Hu G (2013). Activation of NLRP3 inflammasome in alveolar macrophages contributes to mechanical stretch-induced lung inflammation and injury. J Immunol..

[34] Arribas B, Garrido-Mesa N, Peran L, Camuesco D, Comalada M, Bailon E, Olivares M, Xaus J, Kruidenier L, Sanderson IR (2012). The immunomodulatory properties of viable *Lactobacillus salivarius* ssp. salivarius CECT5713 are not restricted to the large intestine. Eur J Nutr.

[35] Wu HJ, Wu E (2012). The role of gut microbiota in immune homeostasis and autoimmunity. Gut Microbes.

[36] Morrow LE, Kollef MH, Casale TB (2010). Probiotic prophylaxis of ventilator-associated pneumonia: a blinded, randomized, controlled trial. Am J Respir Crit Care Med.

[37] Jones RM, Mercante JW, Neish AS (2012). Reactive oxygen production induced by the gut microbiota: pharmacotherapeutic implications. Curr Med Chem.

[38] Ismail AS, Severson KM, Vaishnava S, Behrendt CL, Yu X, Benjamin JL, Ruhn KA, Hou B, DeFranco AL, Yarovinsky F, Hooper LV (2011). Gammadelta intraepithelial lymphocytes are essential mediators of host-microbial homeostasis at the intestinal mucosal surface. Proc Natl Acad Sci USA.

